# 87. Infectious Disease Diversity, Equity, and Antiracism (ID2EA): A Dedicated Curriculum for Infectious Disease Professionals

**DOI:** 10.1093/ofid/ofab466.087

**Published:** 2021-12-04

**Authors:** Shana Gleeson, Meghan Bathgate, Jennifer Frederick, Mahalia S Desruisseaux, Jaimie Meyer, Michael D Virata, Heidi Zapata, Sheela Shenoi, Joanna Radin, Marjorie Golden, Paul Trubin, Albert Shaw, Gerald Friedland, Lydia A Aoun-Barakat

**Affiliations:** 1 Yale School of Medicine, Branford, Connecticut; 2 Yale Poorvu Center for Teaching and Learning, New Haven, Connecticut; 3 Yale University School of Medicine, New Haven, CT; 4 Yale University, New Haven, Connecticut

## Abstract

**Background:**

Systemic bias in the health care system has adverse effects on health outcomes. Educational programs examining the relationship between structural racism and health inequities are needed to translate knowledge into equitable care. The Yale School of Medicine Infectious Disease (ID) Section designed and piloted an innovative *Infectious Disease Diversity, Equity, and Anti-Racism (ID2EA*) curriculum to better understand and confront these issues.

**Methods:**

The ID Section collaborated with pedagogical experts to create a curriculum. A baseline survey of ID faculty and trainees was used to gauge relevant knowledge, attitudes, skills, and topics of interest to participants. The curriculum was designed as a “roadmap” of interactive sessions (“roadmap stops”) focused on topics identified by respondents. Evaluations were performed after events to guide curriculum development and monitor its acceptance and effectiveness.

**Results:**

All respondents (n=28) to the baseline survey agreed that discussion of race and ethnicity should be integrated into medical training. Most respondents (96%) had experience or knowledge of racial microaggressions in the workplace. Fewer (75%) felt comfortable talking to patients about race and only 68% felt confident teaching learners how to decrease bias in care. The survey identified topics of highest priority to participants, including building trust with patients (75%), providing racially sensitive care (68%) and establishing dialogue with community members (57%). Roadmap stops were constructed based on these priorities, with sessions on race-based medical experimentation and inequities, racial segregation and its impact on health, medical mistrust, and a skill building session on improving patient-centered communication. On follow-up surveys (n=18-28), most participants (93%) saw the sessions as a valuable way to spend time and the majority (91%) reported an impact on their understanding of racism in healthcare; specific changes in thinking were qualitatively coded.

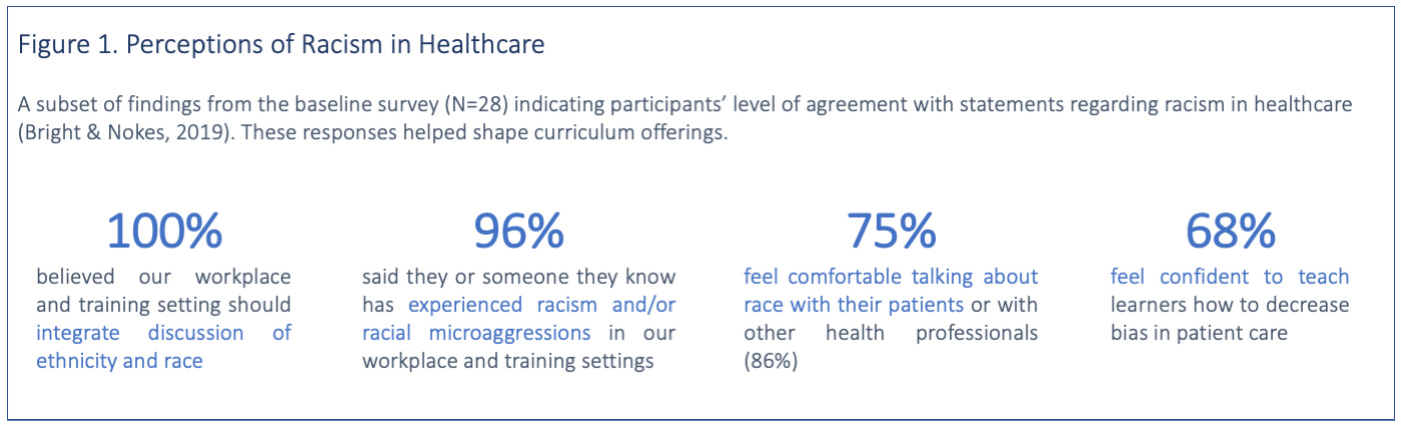

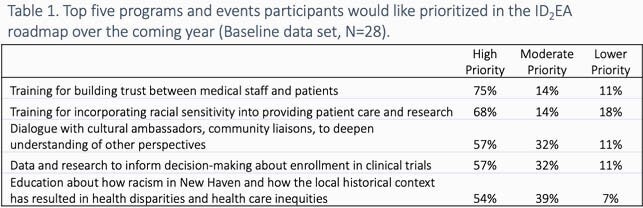

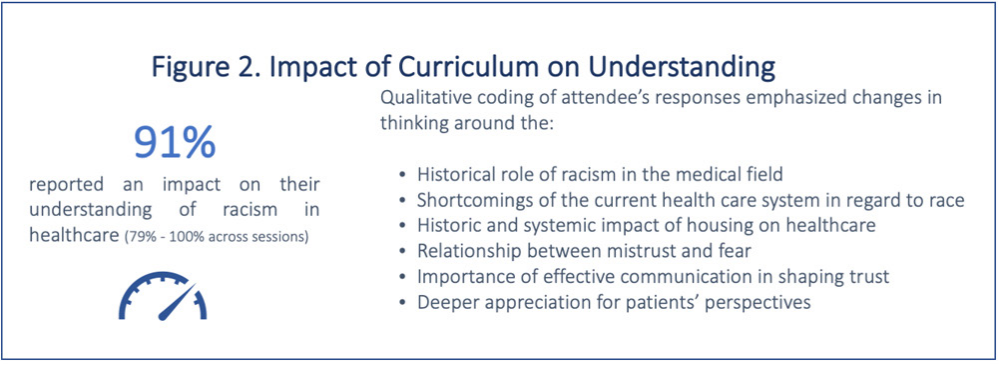

**Conclusion:**

Our findings demonstrate the positive impact of a curriculum to help understand racism and inequities in medicine. Building and implementing a diversity, inclusion, and anti-racism curriculum in ID sections is feasible, beneficial, and valued.

**Disclosures:**

**Jaimie Meyer, MD**, **Gilead Sciences** (Scientific Research Study Investigator) **Sheela Shenoi, MD, MPH**, **Merck** (Other Financial or Material Support, SS’s spouse worked for Merck pharmaceuticals 1997-2007 and retains company stock in his retirement account. There is no conflict of interest, but it is included in the interest of full disclosure.) **Marjorie Golden, MD**, **Iterum Pharmaceuticals** (Consultant)

